# Complementary Effects of Genetic Variations in *LEPR* on Body Composition and Soluble Leptin Receptor Concentration after 3-Month Lifestyle Intervention in Prepubertal Obese Children

**DOI:** 10.3390/nu8060328

**Published:** 2016-05-27

**Authors:** Joanna Gajewska, Alina Kuryłowicz, Ewa Mierzejewska, Jadwiga Ambroszkiewicz, Magdalena Chełchowska, Halina Weker, Monika Puzianowska-Kuźnicka

**Affiliations:** 1 Screening Department, Institute of Mother and Child, Kasprzaka 17a, Warsaw 01-211, Poland; jagoda.ambroszkiewicz@imid.med.pl (J.A.); magdalena.chelchowska@imid.med.pl (M.C.); 2 Department of Human Epigenetics, Mossakowski Medical Research Center, Polish Academy of Sciences, Warsaw 02-106, Poland; kurylowiczala@gazeta.pl (A.K.); mpuzianowska@wum.edu.pl (M.P.-K.); 3 Department of Epidemiology and Biostatistics, Institute of Mother and Child, Warsaw 01-211, Poland; ewa.mierzejewska@imid.med.pl; 4 Department of Nutrition, Institute of Mother and Child, Warsaw 01-211, Poland; halina.weker@imid.med.pl; 5 Department of Geriatrics and Gerontology, Medical Center of Postgraduate Education, Warsaw 01-826, Poland

**Keywords:** leptin receptor, polymorphisms, weight loss, adipokines, prepubertal period

## Abstract

In obese individuals, weight loss might be affected by variants of the adipokine-encoding genes. We verified whether selected functional single nucleotide polymorphisms in *LEP*, *LEPR* and *ADIPOQ* are associated with changes in serum levels of the respective adipokines and weight loss in 100 prepubertal obese (SDS-BMI > 2) Caucasian children undergoing lifestyle intervention. Frequencies of the -2548G > A *LEP*, Q223R *LEPR*, K656N *LEPR*, -11377C > G and -11426A > G *ADIPOQ* polymorphisms were analyzed by restriction fragment length polymorphism. Serum adipokine and soluble leptin receptor (sOB-R) concentrations were measured using the ELISA method. Among the analyzed polymorphisms, only *LEPR* polymorphisms were associated with changes of SDS-BMI or sOB-R concentrations in children after therapy. Carriers of the wild-type K665N and at least one minor Q223R allele had the greatest likelihood of losing weight (OR = 5.09, *p* = 0.006), an increase in sOB-R (*p*_trend_ = 0.022) and decrease in SDS-BMI correlated with the decrease of fat mass (*p* < 0.001). In contrast, carrying of the wild-type Q223R and at least one minor K665N allele were associated with a decrease in sOB-R concentrations and a decrease in SDS-BMI correlated with a decrease in fat-free mass (*p* = 0.002). We suggest that the combination of different LEPR variants, not a single variant, might determine predisposition to weight loss in the prepubertal period.

## 1. Introduction

It is widely recognized that the response to lifestyle interventions, such as changes in dietary habits and physical activity, can be heterogeneous and largely genetically determined [1]. Functional variants of genes involved in appetite regulation, energy metabolism and fat storage are considered candidates to confer susceptibility to weight loss, and their identification may be helpful in the prevention of future health problems. 

Among several genetic variants investigated in relation to the effectiveness of weight loss therapy in obese subjects are Q223R (or Gln223Arg, rs1137101) and K656N (or Lys656Asn, rs8129183) polymorphisms in the leptin receptor gene (*LEPR*) [2,3,4]. Leptin receptor, which is a member of the cytokine receptor family, has several isoforms of different locations and functions [5]. For example, the weight-regulating effects of leptin are mediated by binding to and activation of the specific receptor (long isoform, OB-R_L_) in the brain, while a soluble form of this receptor (one of its short isoforms, sOB-R) modulates leptin levels by binding free leptin in the circulation and preventing the hormone from degradation and clearance [6].

The Q223R A > G (A668G) and K656N G > C (G1968C) polymorphisms in *LEPR* result in nonconservative changes: glutamine to arginine at codon 223 and lysine to asparagine at codon 656, respectively [7,8]. These amino acid substitutions result in changes of the electric charge from neutral to positive for Q223→R and from positive to neutral for K656→N and, therefore, are likely to have functional consequences resulting in changed leptin signaling and, in the broader perspective, an affected response to energy restriction [7]. However, there are only a few studies that have evaluated the impact of these polymorphisms on changes in metabolic parameters during weight loss. 

The diverse response of leptin levels and body mass changes depending on polymorphic variants of *LEP* and *LEPR* has been investigated mainly in obese adults [2,3,9,10,11,12,13]. There are also studies suggesting a gene-gene interaction between the *LEP* and *LEPR* variants in a genetic susceptibility to the development of obesity [14]. A cumulative effect of *LEPR* polymorphisms with other obesity susceptibility loci, including variants in the adiponectin locus (*ADIPOQ*), on weight loss was also observed in Spanish obese adolescents after a multidisciplinary intervention [15]. Case-control studies on the association between polymorphisms in genes encoding adipokines with childhood obesity have been conducted [16,17,18,19,20], but to our knowledge, there is no data concerning the influence of these polymorphisms on weight loss and adipokine concentrations after multidisciplinary intervention. Therefore, the aim of our study was to investigate the effect of selected functional single nucleotide polymorphisms (SNPs) in *LEPR*, *LEP*, and *ADIPOQ* loci on metabolic response and weight loss after a 3-month lifestyle intervention in prepubertal obese children.

## 2. Methods

### 2.1. Subjects

One hundred obese prepubertal Caucasian children aged 5–10 years were recruited between 2010 and 2012 from a group of consecutive patients seeking dietary counseling in the Department of Nutrition at the Institute of Mother and Child in Warsaw. Obesity was classified as standard deviation score-body mass index (SDS-BMI) >2. Exclusion criteria were: (a) presence of endocrine disorders or genetic syndromes, including syndromic obesity; (b) other chronic medical conditions; (c) intake of medications that could affect growth, pubertal development, nutritional or dietary status. Pubertal stage was determined according to the Tanner scale and subjects who showed pubertal development were excluded. Written informed consent was obtained from the parents of all the examined children. The study was performed in accordance with the Helsinki Declaration for Human Research and the study protocol was approved (No. 25/2006) by the Ethics Committee of the Institute of Mother and Child in Warsaw, Poland. 

### 2.2. Dietary Intervention

To evaluate changes in clinical, anthropometric and biochemical parameters in response to lifestyle changes, the obese prepubertal patients underwent a 3-month intervention program consisting of dietary and physical activity modifications and behavioral therapy, including individual psychological care for the child and his/her family. The dietary guidelines, recommending a low-energy diet based on a balanced distribution of carbohydrates, proteins and lipids, for children and their parents, were described in a previous study [21]. The recommended daily energy intake was 1200–1400 kcal/day. The diet was composed of 20% protein, 30% fat and of 50% carbohydrates. Patients had 3–5 meals every day. Study participants did not receive vitamins or mineral supplements. Patients received instructions concerning physical activity, details of which were also previously described [22]. Children were advised to reduce sedentary activities, including watching television and playing computer games to less than two hours a day. Two weeks before the visit in the Department of Nutrition, study participants completed a 10–14 days food diary using a standard questionnaire. Next, randomly selected 3-day records: two consecutive weekdays and one weekend day, before (visit T0) and after 3 months of therapy (visit T3) were analyzed. The subjects and their parents were asked to record the type and amount of food and beverages consumed for the selected days. We evaluated the average daily energy intake and the percentage of energy intake from protein, fat and carbohydrates in the children’s diets. Average daily food rations and their nutritional value were calculated using nutritional analysis software (Dietetyk^®^, National Food and Nutrition Institute, version 2.0, Warsaw, Poland) [23]. The age- and sex-specific dietary reference intake (DRI) percentage was calculated using reference values of daily energy intake for children and adolescents according to Jarosz *et al.* [24]. 

### 2.3. Anthropometric Parameters

Physical examinations, including body height and weight measurements, were performed before (visit T0) and after 3 months of therapy (visit T3). Body height was measured using a standing stadiometer and recorded with a precision of 1 mm. Body weight was assessed unclothed, to the nearest 0.1 kg, with a calibrated balance scale. BMI was calculated as body weight divided by height squared (kg/m^2^). The BMI of each individual was converted to a standard SDS-BMI for the child’s age and sex using Polish reference tables [25]. The data of this reference population were derived from a study concerning the physical development of Warsaw children and adolescents aged 1–18 years, which was conducted in the years 1996–1999 by the Department of Development of Children and Adolescents at the Institute of Mother and Child. Skinfolds were measured with a Holtain skinfold caliper (Holtain Ltd., Wales, UK). Fat mass percentage was calculated according to Lohman’s formula [26].

### 2.4. Biochemical Measurements

Biochemical parameters were determined twice: before (visit T0) and after 3 months of therapy (visit T3). Venous blood samples were collected between 8:00 and 10:00 a.m. after an overnight fast, and centrifuged at 1000× *g* for 10 min at 4 °C. Serum specimens were stored at −70 °C prior to assay. Commercially available ELISA kits (DRG Diagnostics, Marburg, Germany) were used to determine leptin and sOB-R concentrations. Inter-assay variations (CV%) were 5.3% and 3.3% for total leptin and sOB-R, respectively. Serum levels of total adiponectin and high molecular weight (HMW) adiponectin were determined using ELISA kit (ALPCO Diagnostics, Salem, NH, USA). Adiponectin multimers were selectively measured after sample pretreatment with two proteases that specifically digested the trimeric forms or both the hexameric and trimeric forms. In this assay, total adiponectin and HMW adiponectin levels were determined directly. Inter-assay variations were 5.0% and 5.7% for total adiponectin and HMW adiponectin, respectively. Glucose, total cholesterol, LDL and HDL-cholesterol and triglycerides were measured by standard methods (Roche Diagnostics, Basel, Switzerland). To reduce interassay variance, samples obtained before and after therapy were analyzed in one assay.

### 2.5. DNA Isolation and Genetic Analysis

DNA was isolated from 4 mL of venous blood samples by a salting-out procedure [27]. Genotyping of the -2548G > A *LEP* (rs7799039), Q223R (*A*668*G*, rs1137101) and K656N (*G*1968*C*, rs8129183) *LEPR*, as well as -11377C > G (rs266729) and -11426A > G (rs16861194) *ADIPOQ* polymorphisms was performed by PCR amplification followed by digestion with an appropriate restriction enzyme [20]. The obtained restriction fragments were visualized on 2%–3% agarose gels. For quality control of genetic testing: (i) amplification blank controls consisting of only amplification reagents without DNA were performed during each PCR reaction; (ii) randomly selected DNA samples were analyzed by sequencing; (iii) control samples with genotypes identified by sequencing were included into each PCR-RFLP reaction.

### 2.6. Statistical Analysis

The results are presented as means ± standard deviation (SD) for normally distributed data or medians and interquartile range (25th–75th percentiles) for non-normally distributed variables. The Kolmogorov-Smirnov test and graphical inspections of data were used to evaluate the distribution for normality. 

Due to the fact that an increase in height between visit T0 and T3 could be partially responsible for a decrease in SDS-BMI in that time period, weight loss during intervention was defined as SDS-BMI change (Δ) ≤ −0.5. Differences in anthropometric characteristics and biochemical parameters between children who lost weight and those who have not were assessed using the Student t test for normally distributed data and the nonparametric Mann-Whitney test for non-normally distributed data. Paired Student *t* test or nonparametric Wilcoxon test were used to compare the parameters mentioned above before and after intervention in the whole group and in subgroups of children who lost weight and those that did not. 

The Hardy-Weinberg equilibrium analysis was performed using a chi-square test with one degree of freedom. Additive, dominant, recessive and allelic models were tested for every polymorphism for associations with weight loss. Under each model, the odds ratios (ORs) with their 95% confidence intervals (CI) were calculated, and the chi-square test, Fisher exact test and Cochran-Armitage test for trend were used, as appropriate. The procedure described by Benjamini and Hochberg was used to correct *p*-values for multiple testing [28]. Linkage disequilibrium (LD) was analyzed using the pairwise LD measure D’, and haplotype blocks were constructed from population genotype data using Haploview software [29], with the default algorithm for generating haplotype blocks based on methods established by Gabriel *et al.* [30]. A D’ value of 1 indicates complete LD between the two markers; a D’ value greater than 0.8—strong LD; 0.2–0.8—incomplete LD; whereas a D’ of less than 0.2—negligible LD. Multiple logistic regression analysis was conducted to assess the combined impact of two *LEPR* polymorphisms on weight loss during therapy.

Changes in SDS-BMI, body composition and biochemical parameters were expressed as delta variables, calculated by subtracting values at baseline (T0) from values measured after 3 months of therapy (T3). Differences in anthropometric characteristics and biochemical parameters between different genotypes were assessed using the Student *t* test for normally distributed data and the nonparametric Mann-Whitney test for non-normally distributed data. To study associations of the combined genotypes of the *LEPR* polymorphisms Q223R and K656N with changes in soluble leptin receptor concentration, the multivariate linear regression model adjusted for age and sex was used. Genetic variants of the studied polymorphisms were treated as a continuous variable and put in order from the exclusive presence of at least one copy of Q223R minor allele to the exclusive presence of at least one copy of K656N minor allele. Test for linear trend was applied. 

The correlation coefficients between changes in body composition and changes in SDS-BMI due to the intervention in children with combined genotypes of the *LEPR* Q223R and K656N polymorphisms were calculated using Pearson correlation. 

Statistical significance was set at 0.05. Statistical analysis was performed using SPSS v.18.0 software (SPSS Inc., Chicago, IL, USA) and StatXact-3 software, version 3.1 (Cytel Software Corporation, Cambridge, MA, USA).

## 3. Results

### 3.1. Distribution of Polymorphisms in LEPR, LEP and ADIPOQ in Obese Children 

One hundred obese children aged 4–10 years (47% male) were included in the analysis. After 3 months of therapy, mean height and SDS-height in the whole group increased (from 136 cm to 138 cm and from 0.98 to 1.29, respectively, both *p* < 0.001), while median weight, BMI and SDS-BMI decreased (from 47.2 kg to 45.2 kg, 25.1 kg/m^2^ to 23.1 kg/m^2^, and 3.46 to 2.82, respectively, all *p* < 0.001). We categorized the children into two subgroups according to the level of SDS-BMI change during the intervention: with weight loss (ΔSDS-BMI ≤ −0.5, *n* = 71) and without weight loss (ΔSDS-BMI > −0.5, *n* = 29). 

Genotype and allele frequencies of the studied polymorphisms are shown in Table 1. All analyzed genotypes fulfilled the criteria of the Hardy-Weinberg equilibrium. Genotype frequencies of the *LEPR* Q223R (*A* > *G*) polymorphism were significantly different in obese children with and without weight loss. Analysis revealed the strongest association in the dominant genetic model when the presence of the *AG* or the *GG* genotype was associated with more than a three-fold higher likelihood of weight loss (OR = 3.45 (95% CI: 1.38–8.78), *p* = 0.007, adjusted FDR (False Discovery Rate) *p* = 0.035) compared with the *AA* genotype. The frequency of the *G* allele was also higher in obese patients with weight loss than without weight loss (52.1% *vs.* 32.8%; OR = 2.23 (95% CI: 1.18–4.23), *p* = 0.013, adjusted FDR *p* = 0.065). 

We found no differences between the studied groups in genotype and allele distribution for K656N (G > C) *LEPR*, -2548G > A *LEP*, -11377C > G *ADIPOQ* and -11426A > G *ADIPOQ* SNPs.

### 3.2. Associations of Combination of Q223R and K656N LEPR Polymorphisms with Weight Loss in Obese Children after a 3-Month Intervention

Combined genotype frequencies of the *LEPR* Q223R/K656N polymorphisms are shown in Table 2. A complete LD between the two polymorphisms was observed (D’ = 1). The *LEPR* genotype combinations Q223R/K656N: AG/CC, GG/GC and GG/CC were not observed. All children homozygous for one *LEPR* minor allele were wild-type homozygotes for the other.

Based on the finding that the presence of only one minor allele in *LEPR* Q223R was sufficient for a higher likelihood of weight loss, we divided the children into four categories (Table 3): (1) the reference group: wild-type homozygotes for both polymorphisms; (2) subjects with at least one minor allele in Q223R only (genotypes *AG* or *GG*) and wild-type homozygotes for K656N; (3) wild-type homozygotes for Q223R with at least one minor allele in K656N only (genotypes *GC* or *CC*); (4) combined heterozygotes for both loci (Q223R *AG* and K656N *GC*). In the logistic regression model, we confirmed that obese children with at least one minor allele in Q223R and wild-type homozygotes for K656N had a 5-fold higher likelihood of losing weight (ΔSDS-BMI ≤ −0.5) (OR = 5.09 (95% CI: 1.60–16.24), *p* = 0.006) when compared with wild-type homozygotes for both loci. We did not find significant effects of other combinations of *LEPR* polymorphisms on weight loss. 

### 3.3. Body Composition, Biochemical Parameters and Energy Intake before and after Intervention

Of the 100 children enrolled in the study, the full set of biochemical and anthropometric parameters as well as data on nutrition intake before and after intervention were available from 76 (aged 5–10 years, 42.1% male). Genotype and allele frequencies of the *LEPR* Q223R polymorphism in these 76 children were similar to those observed in the whole group. As in the whole group (*n* = 100), it was shown that the subjects carrying at least one minor allele in Q223R polymorphism (the *AG* or *GG* genotype) had a higher likelihood of weight loss (OR = 3.67 (95% CI: 1.24–10.84), *p* = 0.016) than wild-type homozygotes. Moreover, the presence of the G allele (in the allelic model) was associated with a higher probability of ΔSDS-BMI ≤ −0.5 (OR = 2.23 (95% CI: 1.05–4.76), *p* = 0.036) when compared with A allele. 

Both groups of children, who lost weight and those who did not, reduced their energy intake during the therapy (*p* < 0.001) (Table 4). In addition, the proportions of protein, fat and carbohydrates in daily energy intake and percentage of DRI were similar in both groups. Children with and without weight loss at visit T3, at the beginning of the intervention (T0) did not differ significantly in terms of anthropometric parameters, whereas at visit T3 the two groups had a similar height, SDS-height, and SDS-BMI, but significantly different body weight (*p* = 0.037) and BMI (*p* = 0.013). In the obese children with a change in SDS-BMI ≤ −0.5 after the intervention, the median weight decrease was 5% (*p* < 0.001), the median fat mass decrease was 15% (*p* < 0.001), and the mean fat-free mass decrease was 3% (*p* < 0.001), while children with a change in SDS-BMI > −0.5 had similar values of these parameters before and after therapy.

Leptin levels after a 3-month intervention were significantly reduced in both groups, but at visit T3 this parameter was more than 2-fold lower (*p* < 0.001) in patients with weight loss compared with those with a change in SDS-BMI > −0.5 (Table 4). Soluble leptin receptor concentration increased only in children with weight loss (*p* < 0.001) and after the therapy its median concentration was about 30% higher than in those without weight loss (*p* < 0.001). Total adiponectin and HMW-adiponectin levels increased (both *p* < 0.05), while LDL-cholesterol and triglycerides decreased (*p* < 0.05 and *p* = 0.001, respectively) after the therapy only in children who lost weight (Table 4). 

### 3.4. Associations of the LEPR Q223R and K656N Polymorphisms with the Level of Adipokines and Body Composition after 3-Month Intervention

We observed significant differences in the mean decrease in SDS-BMI and percentage of fat mass between individuals carrying different genotypes of the *LEPR* Q223R polymorphism (Table 5). The carriers of at least one minor allele (*AG* heterozygotes and *GG* homozygotes) had significantly greater mean changes of these parameters than the wild-type homozygotes (−0.97 *vs.* −0.63, *p* = 0.01 and −3.3% *vs.* −0.8%, *p* = 0.003, respectively). However, therapy-induced decrease in leptin, sOB-R level, leptin/sOB-R and leptin/adiponectin ratios were similar in the AG + GG and AA groups.

In contrast, we found significant differences in the mean, therapy-induced, decrease of sOB-R level between individuals carrying different genotypes of the *LEPR* K656N polymorphism. The wild-type homozygotes had significantly greater changes of sOB-R than carriers of at least one minor allele (*GC* heterozygotes and *CC* homozygotes), (4.0 ng/mL *vs.* 0.0 ng/mL, *p* = 0.032), despite the fact that therapy-induced changes in body composition were similar in the wild-type homozygotes and carriers of the K656N minor allele (Table 5). 

**Figure 1:**
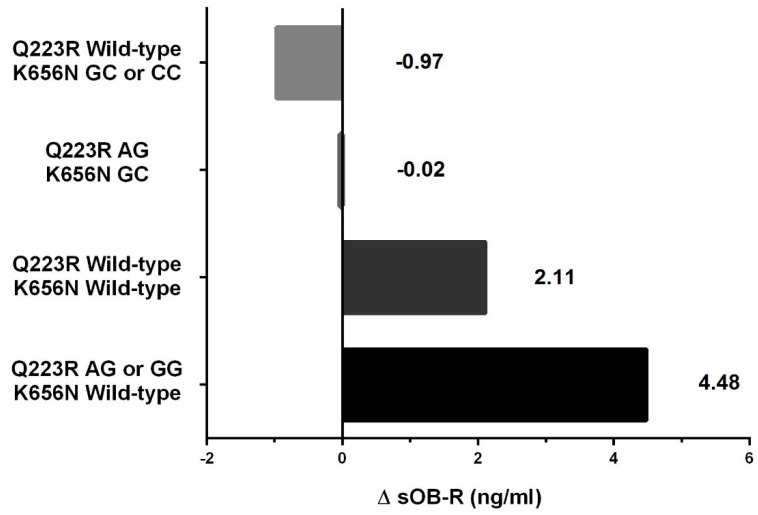
Changes in soluble leptin receptor concentrations in obese children with different genotypes of *LEPR* Q223R and K656N polymorphisms after 3 months of dietary intervention; results of linear regression model adjusted for age and sex.

In the linear regression model, after adjustment for age and sex, the differences in change of sOB-R concentrations among the four groups with different combinations of *LEPR* Q223R and K656N genotypes remained significant (*p*_trend_ = 0.022) with the trend from the highest values in the carriers of at least one minor allele in Q223R only towards lowest values in the carriers of at least one minor allele in K656N only (Figure 1). 

In addition, we found a positive correlation between therapy-induced change in SDS-BMI and change in percentage of fat mass in the carriers of at least one minor allele in Q223R only as well as in the wild-type homozygotes for both polymorphisms (both *p* < 0.001), while we did not observe a significant correlation between changes in SDS-BMI and fat-free mass in these two groups (Table 6). In contrast, in carriers of at least one minor allele in K656N only, a positive correlation between change in SDS-BMI and change in fat-free mass was observed (*p* = 0.002), while no association between change in SDS-BMI and change in percentage of fat mass was found. No correlation between change in SDS-BMI and changes in body composition was found in the combined heterozygotes for both *LEPR* polymorphisms. 

We found no differences in baseline levels and changes during intervention in BMI-SDS, body composition, and adipokine concentrations between carriers of different genotypes of -2548G > A *LEP*, -11377C > G *ADIPOQ* and -11426A > G *ADIPOQ* SNPs. 

## 4. Discussion

Our study shows that both Q223R and K656N polymorphisms in the leptin receptor gene may modulate the effect of lifestyle intervention in obese individuals during the prepubertal period, whereas other investigated SNPs in genes encoding leptin and adiponectin were not related to a reduction in body mass. We also demonstrated that combinations of genotypes of the investigated polymorphisms in *LEPR* may determine predisposition to therapy-induced changes in body composition and soluble leptin receptor concentration in prepubertal children. 

Based on the finding that obese subjects have higher serum levels of leptin and lower levels of its soluble receptor, it has been suggested that human obesity is associated with leptin resistance [31]. Since both the Q223R and the K656N polymorphisms are located within the region encoding the extracellular domain of the leptin receptor, the substitution of one amino acid by another can affect the functional characteristics of the receptor leading to its modified signaling capacity and resulting in various levels of leptin in circulation [1]. 

In our previous work, consistently with the results of other authors, we did not observe an association between these two *LEPR* SNPs and obesity in children and adolescents [20,32,33]. However, none of these studies took into account the combined effect of the Q223R and K656N variants on susceptibility to obesity and the level of serum adipokines. Such an interaction was described by Lu *et al.* [14] who found that a combination of a polymorphism in the *LEP* 3′ flanking region with two polymorphisms in *LEPR* (K109R and K656N) was associated with obesity in a Chinese population, even though none of them individually were associated with body weight or BMI. 

Lifestyle interventions, including modification of dietary habits and increased physical activity, are recommended as an effective therapy for weight reduction as well as for an improvement of biochemical parameters in obese children and adolescents [34]. Like other authors and in accordance with our previous findings, after therapy-induced weight loss we observed a significant decrease in leptin concentration with a parallel increase in soluble leptin receptor and adiponectin levels [35,36,37]. However, about 30% of the studied group did not have a change in body mass or body composition despite having energy intake and percentage of DRI before and after therapy similar to the children with weight loss. This finding implies that response to lifestyle interventions may be genetically determined.

Given the important role of leptin in the regulation of energy metabolism, we suggest that genetic variants of *LEPR* may modulate physiological responses to lifestyle interventions and its influence on health in prepubertal obese children. When analyzed separately, the Q223R and K656N polymorphisms were related to different parameters in children after weight loss therapy: the Q223R *LEPR* SNP was associated with changes in BMI and fat mass percentage, while the K656N with changes in soluble leptin receptor serum concentrations. In contrast, we did not find any differences in serum leptin levels in relation to the studied *LEPR* polymorphisms in obese children before and after intervention. The results of previous studies on the relationship between *LEPR* variants and leptin concentrations in the circulation are ambiguous. Mars *et al.* [10] also demonstrated no effect of the K109R, Q223R and K656N SNPs on an acute decline in leptin levels after only 4 days of energy restriction in men. However, Luis *et al.* [38] observed in Spanish adults a significant decrease in weight, fat mass as well as leptin concentrations in wild-type homozygotes of K656N after a 3months lifestyle intervention, whereas carriers of the minor allele had no decrease in fat mass or leptin levels after this period. In addition, carriers of the minor K656N allele did not respond with a decrease in insulin level or HOMA-IR after weight loss induced by a hypocaloric diet [3]. Similarly to other authors studying the K656N polymorphism in adults, we also suggest that children with the minor allele, which is associated with a lack of soluble leptin receptor changes after therapy, may be more resistant to multidisciplinary intervention than wild-type homozygotes [4]. 

Repasy *et al.* [19] when analyzing the relationship between the Q223R polymorphism and indicators of energy expenditure in obese children (aged 13 ± 2.7 years), also found no association between leptin concentrations and different genotypes of this SNP. However, the wild-type homozygotes compared with other genotypes showed a significantly lower post-absorptive and postprandial respiratory quotient, which could indicate a lower binding capacity of leptin to the soluble form of the receptor in plasma [19]. In our study, we also did not observe any differences in leptin levels between the carriers of the Q223R genotypes before and after therapy in contrast with an adult population in whom differences in leptin concentrations had been observed [39]. These differences could be explained by the younger age of Repasy *et al.*’s [19] study population and our patients, in whom the full effect of Q223R polymorphism has not yet been developed. Nevertheless, our study points at the association between this polymorphism and weight loss in children after lifestyle intervention. Carriers of the Q223R wild-type variant lost significantly less weight and less fat mass after therapy than carriers of the minor allele; therefore, we suggest that wild-type variant carriers may be more resistant to weight loss therapy than carriers of the minor variant. Other authors observed that in adults the wild-type variant of Q223R correlated not only with leptin concentrations but also with higher BMI and body fat, impaired glucose metabolism and dyslipidemia, as well as lower respiratory quotient during low-intensity exercise [40,41]. Therefore, it is suggested that this variant of *LEPR* polymorphism may carry a risk of insulin resistance and liver steatosis in later life. Other genetic variant at the *LEPR* locus (rs3790433) was also associated with increased risk of metabolic syndrome and insulin resistance in adults with metabolic syndrome [12]. In addition, authors found that low plasma level of (*n*-3) and high level of (*n*-6) polyunsaturated fatty acids exacerbated impact of this polymorphism on the risk of hiperinsulinemia and insulin resistance. Many studies have shown that fatty acids influence leptin expression and concentration, but the influence of genetic variations on this is still unknown [42,43]. However, the knowledge of gene-nutrient interactions might be useful in developing personalized dietary recommendations in obese adults as well as obese children. 

To our knowledge, the combined influence of both *LEPR* polymorphisms on soluble leptin receptor levels and weight loss in obese subjects has not yet been analyzed. Our results indicate that the studied *LEPR* polymorphisms may have a complementary effect on the weight loss process in obese prepubertal children during lifestyle intervention. Among different combinations of both *LEPR* SNPs, we found that children with at least one minor allele in Q223R coupled with wild-type K665N had the greatest likelihood of losing weight (5-fold), the greatest increase in soluble leptin receptor concentrations and decrease in SDS-BMI correlated with a decrease in fat mass during intervention. In contrast, carriers of the wild-type Q223R together with at least one minor allele in K665N, presented no difference in likelihood of weight loss when compared with the reference genotype combination. Moreover, only this tested combination showed a tendency towards a decrease in soluble leptin receptor concentration and towards a decrease in SDS-BMI correlated with a decrease in fat-free mass after therapy. It is known that a change of amino acid physico-chemical properties is one of the mechanisms of SNPs affecting protein stability and function, as well as protein-protein interactions [44]. Amino acid substitutions in leptin receptor domain due to SNP in *LEPR* result in changes of the electric charge from neutral to positive for Q223→R and from positive to neutral for K656→N [7]. In our study, in the first combination of both *LEPR* SNPs, mentioned above, resulting in higher odds of weight loss, 223R and K565 were associated with positive electric charge, while in the latter combination, resulting in the lack of the higher likelihood to lose weight, both SNP variants, Q223 and 565N, were associated with neutral electric charge. Therefore, it seems that the consequence of a substitution of one amino acid by another can be a change in leptin-leptin receptor interactions caused by the differences in charge of amino acids, that may alter the efficiency of the weight loss process in children. 

In some studies, functional SNPs in *ADIPOQ* were found to have a significant effect on human body composition, but other reports did not confirm an association between these polymorphisms and obesity [45,46]. In our previous study we found that the -11377C > G *ADIPOQ* polymorphism may modulate the risk of childhood obesity and this polymorphism had a significant impact on the adipokine profile in prepubertal obese children [20]. In the present study, we observed an increase in adiponectin concentrations in obese children with weight loss, while levels of this adipokine remained unchanged in children without weight loss. However, we did not observe any associations between the two analyzed *ADIPOQ* polymorphisms and reduction in body mass in prepubertal children after 3 months of therapy. Sorensen *et al.* [47], when analyzing the -11377C > G *ADIPOQ* SNP in a panel of obesity-related candidate genes in approximately 800 adults, suggested its minor role, if any, in modulating weight changes induced by a moderate hypo-energetic low-fat diet. However, other authors studying 2 SNPs in genes encoding adiponectin (-11377C > G *ADIPOQ*) and its receptor 1 suggested that these polymorphisms may play a role in the responsiveness to dietary fatty acid modification in adults with metabolic syndrome [13]. Several *ADIPOQ* polymorphisms were also studied in Spanish obese adolescents, but none of them were associated with SDS-BMI and fat mass changes after 3 months of multidisciplinary treatment [15], which is in concordance with our results. 

This study has a few potential limitations. Firstly, adiposity was assessed by indirect estimations (BMI, BMI-SDS, skinfold thickness); nevertheless, the same methods were used before and after intervention, so analyzing differences in these parameters should obviate possible measurement errors. Secondly, our findings were obtained from a relatively small sample of subjects, though thoroughly characterized in terms of phenotypes. Therefore, ethnically matched studies should be performed to confirm our results in different populations of prepubertal obese children. Thirdly, the 3-day records provide only fragmentary information about dietary intake. However, these food records were reported to yield the strongest agreement with actual dietary intake compared with 24-h recall and 5-day food frequency records in children [48]. Finally, the lifestyle intervention period in this study was only 3 months and a study of a long-term intervention is needed to verify the relationship between the polymorphisms in adipokine genes and adipokine levels in relation to clinical outcomes.

## 5. Conclusions

We found that out of the 5 analyzed polymorphisms, only the Q223R and K656N in the leptin receptor gene may modulate the weight loss process after lifestyle intervention during the prepubertal period. We also demonstrated a complementary effect of these *LEPR* variants on the predisposition to weight loss, changes in body composition, and to leptin receptor concentrations in obese children. Children with at least one minor allele in Q223R and wild-type K665N had the best likelihood of losing weight which was associated with a decrease in fat mass and an increase in soluble leptin receptor concentrations. We suggest that even though the contribution of a single *LEPR* polymorphism may not be sufficient to influence the weight loss process, the combination of different *LEPR* variants might more precisely determine a child’s predisposition to reduction of body mass in the prepubertal period. 

## Figures and Tables

**Table 1:** Distribution of *LEP*, *LEPR*, and *ADIPOQ* genotypes and alleles in prepubertal obese children with or without weight loss.

Gene/SNP	Obese Children with Weight Loss (ΔSDS-BMI ≤ −0.5) *n* (%)	Obese Children without Weight Loss (ΔSDS-BMI > −0.5) *n* (%)	Inheritance Model	OR (95% CI)	Unadjusted *p*-Value	FDR Adjusted *p*-Value
***LEP* -2548G > A**						
Genotypes						
*GG*	23 (32.4)	10 (34.5)	additive	–	0.767	0.767
*GA*	31 (43.7)	10 (34.5)	dominant	1.10 (0.44, 2.74)	0.840	0.840
*AA*	17 (23.9)	9 (31.0)	recessive	0.70 (0.27, 1.82)	0.463	0.926
Alleles						
G	77 (54.2)	30 (51.7)				
A	65 (45.8)	28 (48.3)	allelic	0.90 (0.49, 1.67)	0.748	0.748
***LEPR* Q223R A > G**						
Genotypes						
*AA*	15 (21.1)	14 (48.3)	additive	–	0.014	0.056
*AG*	38 (53.5)	11 (37.9)	dominant	3.48 (1.38, 8.78)	0.007	**0.035**
*GG*	18 (25.4)	4 (13.8)	recessive	2.12 (0.65, 6.93)	0.205	0.820
Alleles						
A	68 (47.9)	39 (67.2)				
G	74 (52.1)	19 (32.8)	allelic	2.23 (1.18, 4.23)	0.013	0.065
***LEPR* K656N G > C**						
Genotypes						
*GG*	58 (81.7)	19 (65.5)	additive	–	0.206	0.412
*GC*	11 (15.5)	10 (34.5)	dominant	0.43 (0.16, 1.13)	0.081	0.203
*CC*	2 (2.8)	0 (0.0)	recessive	–	1.000	1.000
Alleles						
G	127 (89.4)	48 (82.8)				
C	15 (10.6)	10 (17.2)	allelic	0.57 (0.24, 1.35)	0.195	0.488
***ADIPOQ* -11426A > G**						
Genotypes						
*AA*	59 (83.1)	23 (79.3)	additive	–	–	
*AG*	12 (16.9)	6 (20.7)	dominant	0.78 (0.26, 2.32)	0.655	1.092
*GG*	0 (0.0)	0 (0.0)	recessive	–	–	
Alleles						
A	130 (91.5)	52 (89.7)				
G	12 (8.5)	6 (10.3)	allelic	0.80 (0.28, 2.24)	0.671	0.839
***ADIPOQ* -11377C > G**						
Genotypes						
*CC*	31 (43.7)	12 (41.4)	additive	–	0.657	0.876
*CG*	31 (43.7)	12 (41.4)	dominant	0.91 (0.38, 2.18)	0.834	1.043
*GG*	9 (12.6)	5 (17.2)	recessive	0.70 (0.21, 2.29)	0.540	0.720
Alleles						
C	93 (65.5)	36 (62.1)				
G	49 (34.5)	22 (37.9)	allelic	0.86 (0.46, 1.62)	0.646	1.077

FDR, false discovery rate; OR, odds ratio; BMI, body mass index; SDS, standard deviation score; SNP, single nucleotide polymorphism; CI, confidence interval.

**Table 2:** Distribution of Q223R and K656N *LEPR* genotypes in prepubertal obese children.

		LEPR K656N *n* (%)			Total
		*GG*	*GC*	*CC*	
**LEPR Q223R**	***AA***	18 (18.0%)	9 (9.0%)	2 (2.0%)	29.0%
	***AG***	37 (37.0%)	12 (12.0%)	–	49.0%
	***GG***	22 (22.0%)	–	–	22.0%
	Total	77.0%	21.0%	2.0%	100.0%

**Table 3:** Logistic regression model of the combined influence of Q223R and K656N *LEPR* polymorphisms on weight loss (ΔSDS-BMI ≤ −0.5) in prepubertal obese children.

Variable	Children with Weight Loss (ΔSDS-BMI ≤ −0.5) *n* (%)	Children without Weight Loss (ΔSDS-BMI > −0.5) *n* (%)	OR	95% CI	*p*
Q223R/K656N genotypes					
Wild-type/Wild-type	9 (12.7%)	9 (31%)	Ref	-	-
*AG* or *GG*/Wild-type	49 (69.0%)	10 (34.5%)	5.09	(1.60, 16.24)	0.006
Wild-type/*GC* or *CC*	6 (8.5%)	5 (17.2%)	1.19	(0.25, 5.60)	0.828
*AG*/*GC*	7 (9.9%)	5 (17.2%)	1.53	(0.34, 6.85)	0.580
Sex					
Male	35 (49.3%)	12 (41.4%)	Ref	-	-
Female	36 (50.7%)	17 (58.6%)	0.95	(0.36, 2.51)	0.912
Age (year)	–		0.85	(0.62, 1.16)	0.301

OR, odds ratio; CI, confidence interval; Ref, reference.

**Table 4:** Clinical and biochemical characteristics and dietary intake in prepubertal obese children with and without weight loss.

	Obese Children with Weight Loss (ΔSDS-BMI ≤ −0.5) *n* = 56			Obese Children without Weight Loss (ΔSDS-BMI > −0.5) *n* = 20			*p* (T0 *vs.* T0)	*p* (T3 *vs.* T3)
	T0	T3	*p* (T3 *vs.* T0)	T0	T3	*p* (T3 *vs.* T0)		
Age (years)	8.1 (6.8–9.2)		-	8.8 (7.3–9.6)		-	0.110	
Male (%)	44.6		-	35.0		-	0.453	
**Anthropometric parameters**								
Height (cm)	135 ± 10	137 ± 10	<0.001	139 ± 10	141 ± 10	<0.001	0.137	0.152
SDS-height	0.99 ± 1.01	1.31 ± 1.00	<0.001	1.14 ± 1.08	1.42 ± 1.12	<0.001	0.572	0.700
Weight (kg)	45.2 (39.6–56.5)	42.9 (35.7–51.8)	<0.001	50.7 (39.7–56.6)	50.8 (39.8–56.3)	0.779	0.501	**0.037**
BMI	24.7 (23.1–28.1)	22.6 (20.4–25.5)	<0.001	25.8 (23.5–27.0)	25.2 (22.8–26.8)	0.002	0.929	**0.013**
SDS-BMI	3.52 (2.71–4.81)	2.41 (1.73–3.89)	<0.001	3.40 (2.88–4.02)	3.10 (2.50–3.87)	0.002	0.596	0.081
**Body composition**								
Fat mass (%)	41.1 (39.3–44.5)	38.4 (33.9–41.5)	<0.001	39.6 (36.7–43.4)	40.3 (37.2–43.5)	0.911	0.183	0.253
Fat mass (kg)	19.9 (15.7–23.7)	16.8 (13.5–20.9)	<0.001	20.8 (16.9–22.9)	20.3 (16.1–24.3)	0.881	0.832	0.031
Fat-free mass (kg)	27.5 ± 6.9	26.8 ± 6.5	<0.001	30.3 ± 6.8	30.3 ± 6.7	0.876	0.130	0.048
**Biochemical measurements**								
Leptin (ng/mL)	37.0 (23.5–54.7)	11.8 (5.9–26.6)	<0.001	42.9 (29.3–66.2)	26.5 (15.1–45.0)	0.004	0.288	0.001
Soluble leptin receptor (ng/mL)	23.0 (18.2–26.8)	28.4 (23.2–31.7)	<0.001	22.8 (18.7–26.2)	21.5 (19.3–24.1)	0.444	0.911	0.000
Leptin/soluble leptin receptor	1.51 (0.88–2.92)	0.46 (0.19–0.98)	<0.001	1.85 (1.18–2.67)	1.33 (0.65–2.24)	0.030	0.509	0.000
Total adiponectin (μg/mL)	6.6 (5.0–7.4)	7. 0 (5.8–8.3)	0.046	5.7 (4.4–6.5)	6.9 (4.5–7.7)	0.332	0.135	0.233
HMW adiponectin (μg/mL)	3.3 (2.1–4.5)	3.9 (3.0–4.7)	0.001	2.9 (2.1–4.1)	3.1 (2.0–4.9)	0.687	0.615	0.166
HMW/total adiponectin (%)	49.0 ± 14.4	53.5 ± 12.3	0.010	53.1 ± 14.8	49.9 ± 17.1	0.394	0.319	0.353
Leptin/adiponectin	6.26 (3.23–9.69)	1.87 (0.91-4.09)	<0.001	8.79 (5.34–12.02)	4.22 (3.49–7.87)	0.003	0.104	0.001
Glucose (mg/dL)	84 ± 6	82 ± 6	0.087	83 ± 4	84 ± 5	0.255	0.475	0.190
Total cholesterol (mg/dL)	154 (144–165)	152 (140–165)	0.117	156 (145–163)	158 (139–176)	0.478	0.591	0.224
HDL-cholesterol (mg/dL)	49 (42–60)	50 (41–57)	0.763	48 (39–54)	53 (43–61)	0.190	0.425	0.439
LDL-cholesterol (mg/dL)	95 (85–99)	89 (76–101)	0.028	91 (83–103)	95 (79–113)	0.260	0.671	0.278
Triglycerides (mg/dL)	91 (69–115)	75 (49–96)	0.001	93 (58–135)	82 (58–117)	0.601	0.962	0.330
**Dietary intake**								
Energy (kcal/24 h)	1727 (1431–2136)	1215 (962–1369)	<0.001	1822 (1493–2080)	1278 (1018–1546)	<0.001	0.972	0.305
Energy (% of DRI)	93.9 (78.5–112.5)	63.4 (49.0–73.3)	<0.001	82.1 (72.1–113.8)	55.4 (41.3–78.1)	<0.001	0.288	0.716
Proteins (% of energy intake)	13.5 (12.3–15.7)	16.8 (15.1–18.6)	<0.001	13.3 (12.7–15.3)	16.1 (14.0–19.1)	0.009	0.732	0.508
Carbohydrates (% of energy intake)	53.4 (47.7–55.9)	51.2 (48.2–55.5)	0.956	52.3 (47.8–56.1)	51.3 (49.4–55.5)	0.936	0.906	0.928
Fat (% of energy intake)	33.5 (31.0–38.0)	30.9 (28.7–34.0)	0.008	35.1 (31.2–37.3)	33.3 (27.5–38.2)	0.376	0.813	0.406
Proteins (% of DRI)	219 (170–266)	172 (142–210)	<0.001	201 (160–270)	169 (123–195)	0.006	0.436	0.516
Carbohydrates (% of DRI)	337 (285–428)	231 (189–287)	<0.001	312 (277–414)	257 (199–300)	<0.001	0.870	0.716
Fat (% of DRI)	104 (90–136)	71 (53–82)	<0.001	111 (83–144)	70 (53–102)	0.001	0.870	0.428

Results are presented as means ± standard deviations for normally distributed data, or medians and interquartile ranges (25th–75th percentiles) for non-normally distributed variables; SDS: standard deviation score, BMI: body mass index, HMW: high molecular weight, DRI: dietary reference intake.

**Table 5:** Associations of *LEPR* Q223R and K656N polymorphisms with level of adipokines and body composition before and after 3-month intervention.

	LEPR Q223R A > G			LEPR K656N G > C		
	Q223 (*AA*) (*n* = 22)	223R (*AG* + *GG*) (*n* = 54)	*p AA vs. AG* + *GG*	K656 (*GG*) (*n* = 60)	656N (*GC* + *CC*) (*n* = 16)	*p GG vs. GC* + *CC*
Baseline SDS-BMI	3.52 (3.11–4.12)	3.32 (2.60–5.05)	0.630	3.48 (2.65–4.75)	3.40 (2.96–5.74)	0.495
Delta SDS-BMI	−0.63 ± 0.53	−0.97 ± 0.50	0.010	−0.89 ± 0.53	−0.79 ± 0.55	0.494
Baseline fat mass (%)	40.2 (37.3–44.0)	41.1 (38.9–44.3)	0.464	40.7 (37.5–43.8)	42.2 (37.9–44.2)	0.499
Delta fat mass (%)	−0.8 ± 2.9	-3.3 ± 3.2	0.003	−2.9 ± 3.4	-1.2 ± 2.7	0.068
Baseline fat-free mass (kg)	29.7 ± 5.8	27.7 ± 7.4	0.114	28.2 ± 6.9	28.4 ± 7.3	0.819
Delta fat-free mass (kg)	−0.7 ± 1.1	−0.4 ± 1.2	0.402	−0.4 ± 1.2	-0.8 ± 1.0	0.342
Baseline Leptin (ng/mL)	44.0 (28.3–65.9)	37.0 (22.8–54.6)	0.166	37.0 (21.5–55.1)	46.0 (32.4–61.4)	0.125
Delta Leptin (ng/mL)	−26.6 ± 24.4	−21.2 ± 20.5	0.334	−22.2 ± 21.4	−24.9 ± 23.2	0.670
Baseline sOB-R (ng/mL)	22.0 (17.6–27.6)	23.1 (19.6–26.6)	0.639	23.1 (18.2–26.5)	22.3 (19.3–28.3)	0.828
Delta sOB-R (ng/mL)	1.2 ± 6.3	3.9 ± 6.7	0.108	4.0 ± 6.4	0.0 ± 6.9	0.032
Baseline Leptin/sOB-R	2.24 (1.09–3.29)	1.50 (0.89–2.50)	0.204	1.42 (.86–2.69)	2.27 (1.43–3.02)	0.198
Delta Leptin/sOB-R	−0.82 (−2.52–−0.15)	−0.85 (−1.68–−0.36)	0.891	−0.82 (-1.94–−0.34)	−1.02 (−2.07–−0.09)	0.674
Baseline Leptin/adiponectin	7.79 (5.37–11.86)	6.05 (3.32–9.98)	0.248	6.63 (3.34–10.63)	7.06 (5.13–10.98)	0.333
Delta Leptin/adiponectin	−4.5 ± 3.7	−4.0 ± 3.8	0.628	−4.22 ± 3.62	−3.72 ± 4.36	0.678

Results are presented as means ± standard deviations for normally distributed data, or medians and interquartile ranges (25th–75th percentiles) for non-normally distributed variables; SDS: standard deviation score, BMI: body mass index, sOB-R: soluble leptin receptor.

**Table 6:** Correlations of changes in fat mass and fat-free mass with change in SDS-BMI in children with different genotypes of Q223R and K656N *LEPR* polymorphisms after 3 months of dietary intervention.

Q223R/K656N Genotype	Change in Fat Mass (%)		Change in Fat-Free Mass (kg)	
	Pearson *r*	*p*	Pearson *r*	*p*
Wild-type/Wild-type	0.858	<0.001	0.334	0.244
*AG* or *GG*/Wild-type	0.519	<0.001	0.194	0.197
Wild-type/*GC* or *CC*	0.372	0.364	0.904	0.002
*AG*/*GC*	0.566	0.144	0.683	0.062

## References

[1] Ghalandari H., Hosseini-Esfahani F., Mirmiran P. (2015). The association of polymorphisms in leptin/leptin receptor genes and ghrelin/ghrelin receptor genes with overweight/obesity and the related metabolic disturbances: A review. Int. J. Endocrinol. Metab..

[2] De Luis Roman D., Aller R., Izaola O., Sagrado M.G., Conde R. (2008). Influence of Lys656Asn polymorphism of leptin receptor gene on leptin response secondary to two hypocaloric diets: A randomized clinical trial. Ann. Nutr. Metab..

[3] De Luis Roman D., Aller R., Izaola O., Gonzalez Sagrado M., Conde R., de la Fuente B., Primo D. (2015). Effect of Lys656Asn polymorphism of leptin receptor gene on cardiovascular risk factors and serum adipokine levels after a high polyunsaturated fat diet in obese patients. J. Clin. Lab. Anal..

[4] Rudkowska I., Pérusse L. (2012). Individualized weight management: What can be learned from nutrigenomics and nutrigenetics?. Prog. Mol. Biol. Transl. Sci..

[5] Farooqi I.S., Wangensteen T., Collins S., Kimber W., Matarese G., Keogh J.M., Lank E., Bottomley B., Lopez-Fernandez J., Ferraz-Amaro I. (2007). Clinical and molecular genetic spectrum of congenital deficiency of the leptin receptor. N. Engl. J. Med..

[6] Zastrow O., Seidel B., Kiess W., Thiery J., Keller E., Bottuer A., Kratzsch J. (2003). The soluble leptin receptor is crucial for leptin action: Evidence from clinical and experimental data. Int. J. Obes. Relat. Metab. Disord..

[7] Chung W.K., Power-Kehoe L., Chua M., Chu F., Aronne L., Huma Z., Sothern M., Udall J.N., Kahle B., Leibel R.L. (1997). Exonic and intronic sequence variation in the human leptin receptor gene (LEPR). Diabetes.

[8] Richert L., Chevalley T., Manen D., Bonjour J.P., Rizzoli R., Ferrari S. (2007). Bone mass in prepubertal boys is associated with a Gln223Arg amino acid substitution in the leptin receptor. J. Clin. Endocrinol. Metab..

[9] Lakka T.A., Rankinen T., Weisnagel S.J., Chagnon Y.C., Lakka H.M., Ukkola O., Boulé N., Rice T., Leon A.S., Skinner J.S. (2004). Leptin and leptin receptor gene polymorphisms and changes in glucose homeostasis in response to regular exercise in nondiabetic individuals: The HERITAGE family study. Diabetes.

[10] Mars M., van Rossum C.T., de Graaf C., Hoebee B., de Groot L.C., Kok F.J. (2004). Leptin responsiveness to energy restriction: Genetic variation in the leptin receptor gene. Obes. Res..

[11] Bašić M., Butorac A., Landeka Jurčević I., Bačun-Družina V. (2012). Obesity: Genome and environment interactions. Arh. Hig. Rada Toksikol..

[12] Phillips C.M., Goumidi L., Bertrais S., Field M.R., Ordovas J.M., Cupples L.A., Defoort C., Lovegrove J.A., Drevon C.A., Blaak E.E. (2010). Leptin receptor polymorphisms interact with polyunsaturated fatty acids to augment risk of insulin resistance and metabolic syndrome in adults. J. Nutr..

[13] Ferguson J.F., Phillips C.M., Tierney A.C., Pérez-Martínez P., Defoort C., Helal O., Lairon D., Planells R., Shaw D.I., Lovegrove J.A. (2010). Gene-nutrient interactions in the metabolic syndrome: Single nucleotide polymorphisms in ADIPOQ and ADIPOR1 interact with plasma saturated fatty acids to modulate insulin resistance. Am. J. Clin. Nutr..

[14] Lu J., Zou D., Zheng L., Chen G., Lu J., Feng Z. (2013). Synergistic effect of LEP and LEPR gene polymorphism on body mass index in a Chinese population. Obes. Res. Clin. Pract..

[15] Moleres A., Rendo-Urteaga T., Zulet M.A., Marcos A., Campoy C., Garagorri J.M., Martínez J.A., Azcona-Sanjulián M.C., Marti A. (2012). Obesity susceptibility loci on body mass index and weight loss in Spanish adolescents after a lifestyle intervention. J. Pediatr..

[16] Petrone A., Zavarella S., Caiazzo A., Leto G., Spoletini M., Potenziani S., Osborn J., Vania A., Buzzetti R. (2006). The promoter region of the adiponectin gene is a determinant in modulating insulin sensitivity in childhood obesity. Obesity.

[17] Tabassum R., Mahendran Y., Dwivedi O.P., Chauhan G., Ghosh S., Marwaha R.K., Tandon N., Bharadwaj D. (2012). Common variants of IL6, LEPR, and PBEF1 are associated with obesity in Indian children. Diabetes.

[18] León-Mimila P., Villamil-Ramírez H., Villalobos-Comparán M., Villarreal-Molina T., Romero-Hidalgo S., López-Contreras B., Gutiérrez-Vidal R., Vega-Badillo J., Jacobo-Albavera L., Posadas-Romeros C. (2013). Contribution of common genetic variants to obesity and obesity-related traits in Mexican children and adults. PLoS ONE.

[19] Répásy J., Bokor S., Erhardt É., Molnár D. (2014). Association of Gln223 Arg polymorphism of the leptin receptor gene with indicators of energy expenditure in obese children. Nutrition.

[20] Gajewska J., Kuryłowicz A., Ambroszkiewicz J., Mierzejewska E., Chełchowska M., Szamotulska K., Weker H., Puzianowska-Kuźnicka M. (2016). ADIPOQ -11377C > G polymorphism increases the risk of adipokine abnormalities and child obesity regardless of dietary intake. J. Pediatr. Gastroenterol. Nutr..

[21] Weker H. (2006). Simple obesity in children. A study on the role of nutritional factors. Med. Wieku Rozwoj..

[22] Oblacińska A., Weker H. (2008). Prevention of Obesity in Children and Adolescents.

[23] Dzieniszewski J., Szponar L., Szczygieł B., Socha J. (2001). Scientific Foundations of Nutrition in Hospitals in Poland.

[24] Jarosz M., Traczyk I., Rychlik E., Jarosz M. (2012). Energia. Normy Żywienia dla Populacji Polskiej—Nowelizacja.

[25] Palczewska I., Niedzwiedzka Z. (2001). Somatic development indices in children and youth of Warsaw. Med. Wieku Rozwoj..

[26] Lohman T.G. (1981). Skinfolds and body density and their relation to body fatness: A review. Hum. Biol..

[27] Miller S.A., Dykes D.D., Polesky H.F. (1988). A simple salting out procedure for extracting DNA from human nucleated cells. Nucleic Acids Res..

[28] Benjamini Y., Hochberg Y. (1995). Controlling the false discovery rate: A practical and powerful approach to multiple testing. J. R. Stat. Soc. B.

[29] Barrett J.C., Fry B., Maller J., Daly M.J. (2005). Haploview: Analysis and visualization of LD and haplotype maps. Bioinformatics.

[30] Gabriel S.B., Schaffner S.F., Nguyen H., Moore J.M., Roy J., Blumenstiel B., Higgins J., DeFelice M., Lochner A., Faggart M. (2002). The structure of haplotype blocks in the human genome. Science.

[31] Xu S., Xue Y. (2016). Pediatric obesity: Causes, symptoms, prevention and treatment. Exp. Ther. Med..

[32] Zandoná M.R., Rodrigues R.O., Albiero G., Campagnolo P.D., Vitolo M.R., Almeida S., Mattevi V.S. (2013). Polymorphisms in LEPR, PPARG and APM1 genes: Associations with energy intake and metabolic traits in young children. Arq. Bras. Endocrinol. Metab..

[33] Komşu-Ornek Z., Demirel F., Dursun A., Ermiş B., Pişkin E., Bideci A. (2012). Leptin receptor gene Gln223Arg polymorphism is not associated with obesity and metabolic syndrome in Turkish children. Turk. J. Pediatr..

[34] Ho M., Garnett S.P., Baur L., Burrows T., Stewart L., Neve M., Collins C. (2012). Effectiveness of lifestyle interventions in child obesity: Systematic review with meta-analysis. Pediatrics.

[35] Reinehr T., Roth C., Menke T., Andler W. (2004). Adiponectin before and after weight loss in obese children. J. Clin. Endocrinol. Metab..

[36] Cambuli V.M., Musiu M.C., Incani M., Paderi M., Serpe R., Marras V., Cossu E., Cavallo M.G., Mariotti S., Loche S. (2008). Assessment of adiponectin and leptin as biomarkers of positive metabolic outcome after lifestyle intervention in overweight and obese children. J. Clin. Endocrinol. Metab..

[37] Gajewska J., Weker H., Ambroszkiewicz J., Szamotulska K., Chełchowska M., Franek E., Laskowska-Klita T. (2013). Alterations in markers of bone metabolism and adipokines following a 3-month lifestyle intervention induced weight loss in obese prepubertal children. Exp. Clin. Endocrinol. Diabetes.

[38] De Luis Roman D., de la Fuente R.A., Sagrado M.G., Izaola O., Vicente R.C. (2006). Leptin receptor Lys656Asn polymorphism is associated with decreased leptin response and weight loss secondary to a lifestyle modification in obese patients. Arch. Med. Res..

[39] Quinton N.D., Lee A.J., Ross R.J., Eastell R., Blakemore A.I. (2001). A single nucleotide polymorphism (SNP) in the leptin receptor is associated with BMI, fat mass and leptin levels in postmenopausal Caucasian women. Hum. Genet..

[40] Furusawa T., Naka I., Yamauchi T., Natsuhara K., Kimura R., Nakazawa M., Ishida T., Inaoka T., Matsumura Y., Ataka Y. (2010). The Q223R polymorphism in LEPR is associated with obesity in Pacific Islanders. Hum. Genet..

[41] Loos R.J., Rankinen T., Chagnon Y., Tremblay A., Pérusse L., Bouchard C. (2006). Polymorphisms in the leptin and leptin receptor genes in relation to resting metabolic rate and respiratory quotient in the Québec Family Study. Int. J. Obes..

[42] Reseland J.E., Syversen U., Bakke I., Qvigstad G., Eide L.G., Hjertner O., Gordeladze J.O., Drevon C.A. (2001). Leptin is expressed in and secreted from primary cultures of human osteoblasts and promotes bone mineralization. J. Bone. Miner. Res..

[43] Lombardo Y.B., Hein G., Chicco A. (2007). Metabolic syndrome: Effects of *n*-3 PUFAs on a model of dyslipidemia, insulin resistance and adiposity. Lipids.

[44] Kucukkal T.G., Petukh M., Li L., Alexov E. (2015). Structural and physico-chemical effects of disease and non-disease nsSNPs on proteins. Curr. Opin. Struct. Biol..

[45] An S.S., Hanley A.J., Ziegler J.T., Brown W.M., Haffner S.M., Norris J.M., Rotter J.I., Guo X., Chen Y.D., Wagenknecht L.E. (2012). Association between ADIPOQ SNPs with plasma adiponectin and glucose homeostasis and adiposity phenotypes in the IRAS Family Study. Mol. Genet. Metab..

[46] Karmelic I., Lovric J., Bozina T., Ljubić H., Vogrinc Ž., Božina N., Sertić J. (2012). Adiponectin level and gene variability are obesity and metabolic syndrome markers in young population. Arch. Med. Res..

[47] Sørensen T.I., Boutin P., Taylor M.A., Larsen L.H., Verdich C., Petersen L., Holst C., Echwald S.M., Dina C., Toubro S. (2006). Genetic polymorphisms and weight loss in obesity: A randomised trial of hypo-energetic high- *versus* low-fat diets. PLoS Clin. Trials.

[48] Crawford P.B., Obarzanek E., Morrison J., Sabry Z.I. (1994). Comparative advantage of 3-day food records over 24-h recall and 5-day food frequency validated by observation of 9- and 10-year-old girls. J. Am. Diet. Assoc..

